# Giant Osteolipoma Fixed to the Greater Trochanter of the Femur in a Seventy-Year-Old Elderly Woman

**DOI:** 10.7759/cureus.1036

**Published:** 2017-02-17

**Authors:** Ali J Electricwala, Yogesh Panchwagh, Jaffer T Electricwala

**Affiliations:** 1 Department of Orthopaedics, Electricwala Hospital and Clinic; 2 Orthopaedic Oncology Clinic, Sancheti Hospital

**Keywords:** giant, osteolipoma, greater trochanter

## Abstract

A lipoma containing mature osseous elements is called osteolipoma. This article describes a giant osteolipoma fixed to the periosteum of the greater trochanter of the femur. A seventy-year-old woman presented with a large subcutaneous mass in the right buttock which had been present for six years. On local examination, a giant mass that was ovoid, firm, non-tender, well demarcated, subcutaneous, and relatively fixed to the greater trochanter was palpated in the right buttock. A medical imaging and fluoro-deoxy-glucose (FDG) bone scan revealed a large lipomatous and metabolically active lesion arising from the periosteum of the greater trochanter of femur. The excisional mass of 17 × 8 × 7 cm^3^ was well encapsulated and had an osseous basal portion. Cut sections of the mass revealed mainly yellow fatty tissue surrounded by a thin fibrous capsule with numerous interlacing thin lamellar bony structures. The definitive pathological diagnosis was osteolipoma without evidence of malignancy. No recurrence was observed at eight months follow-up.

Osteolipoma with an osseous basal portion is rare. Surgical excision is the treatment of choice and the prognosis is good. To the best of our knowledge, this is the first report of an unusual giant osteolipoma fixed to the periosteum of the greater trochanter of the femur.

## Introduction

Lipomas are the most common benign soft tissue tumors and can occur anywhere in the body [1]. When a lipoma independent of bone undergoes ossification, it is called osteolipoma. The terms ossifying lipoma, osseous lipoma, and lipoma with osseous metaplasia have been used interchangeably with osteolipoma [2-3]. In this case, we report an unusual giant osteolipoma fixed to the periosteum of the greater trochanter of the femur.

Written informed consent was obtained from the patient for the publication of this case report and accompanying images.

## Case presentation

A seventy-year-old woman presented to the authors with a painful mass in the right buttock. The patient noted the swelling six years back, which was insidious in onset and gradually progressive in nature, but she did not bring it to the attention of a physician because it caused only slight discomfort while sitting. She had no family history and had not suffered any severe trauma. Two weeks prior to presentation, her discomfort became obvious and she began experiencing pain while sitting and walking. The pain was dull-aching in nature and did not radiate to other regions. On routine physical examination, she appeared healthy with no known medical comorbidity. Her gait was normal.

On local examination, a giant mass that was ovoid, firm, non-tender, well demarcated, subcutaneous, and relatively fixed to the greater trochanter was palpated in the right buttock. The hip and knee movements were normal. The neurological examination was within normal limits, and no lymphadenopathy was present.

**Figure 1:**
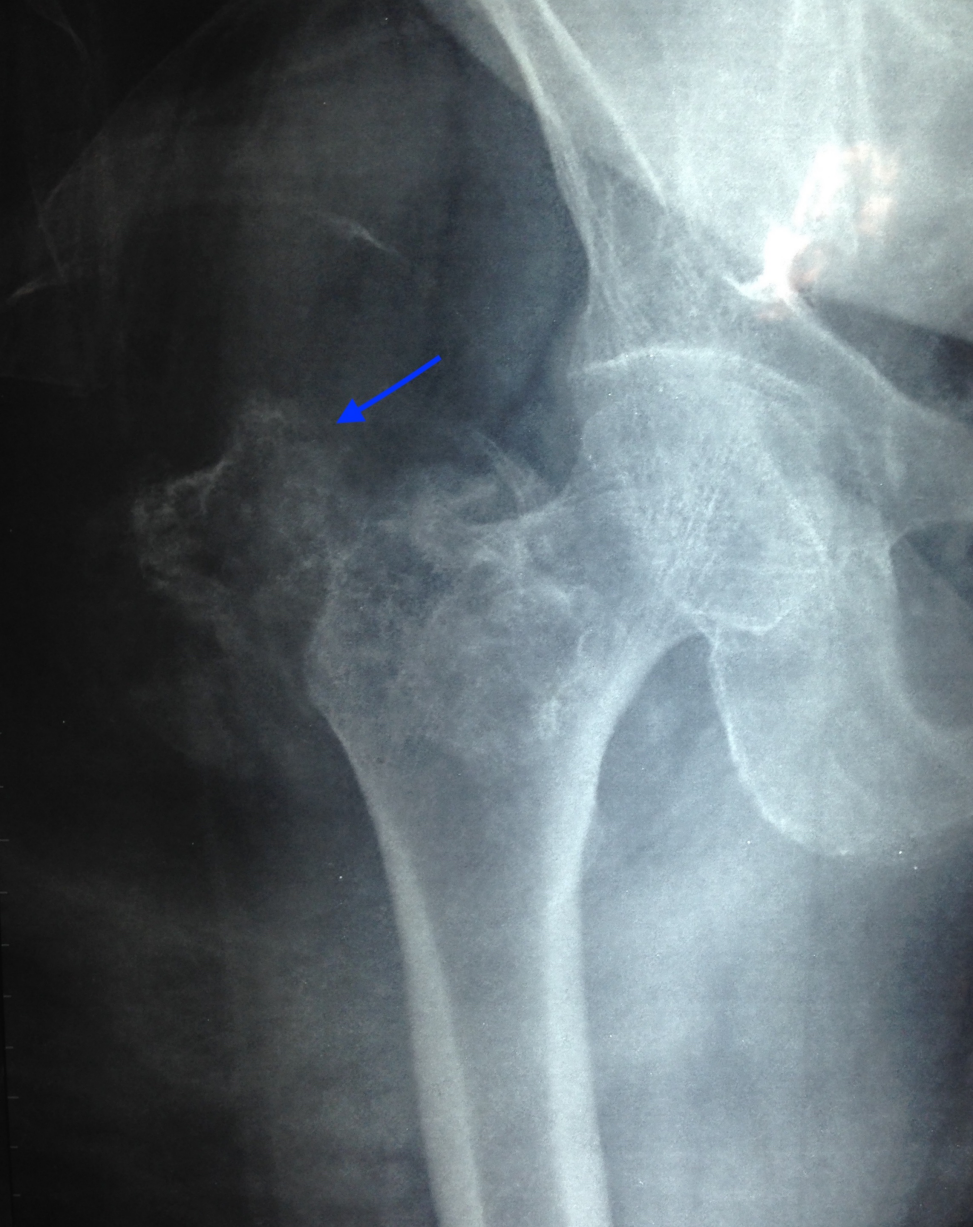
A plain X-ray showing dispersed calcification close to the periosteum of the greater trochanter of the femur

Her laboratory parameters were normal except for a raised erythrocyte sedimentation rate (36 mm/hour). A plain X-ray revealed dispersed calcification close to the periosteum of the greater trochanter of the femur (Figure 1).

**Figure 2:**
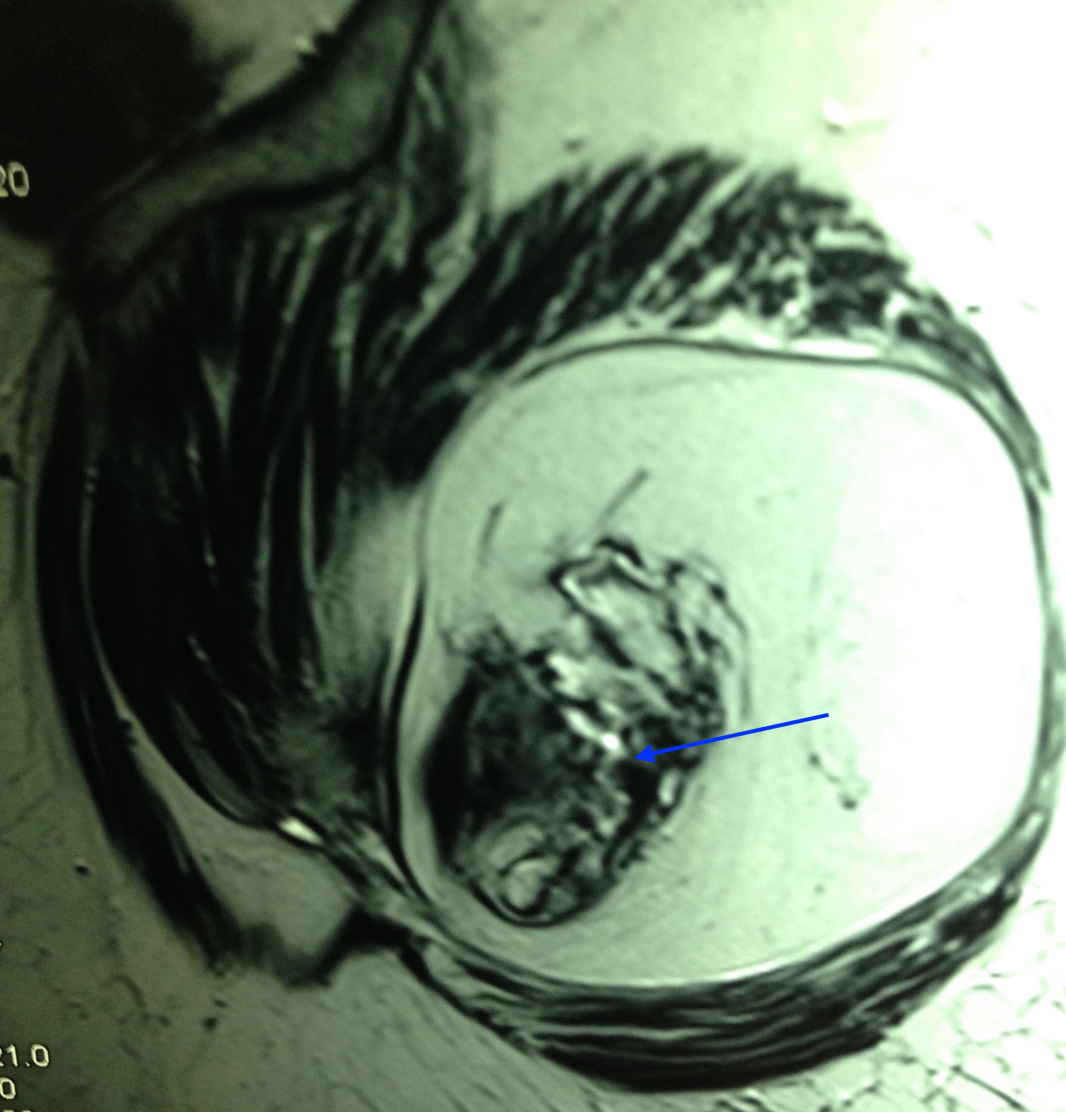
T1-weighted MRI showing tumour mass

**Figure 3:**
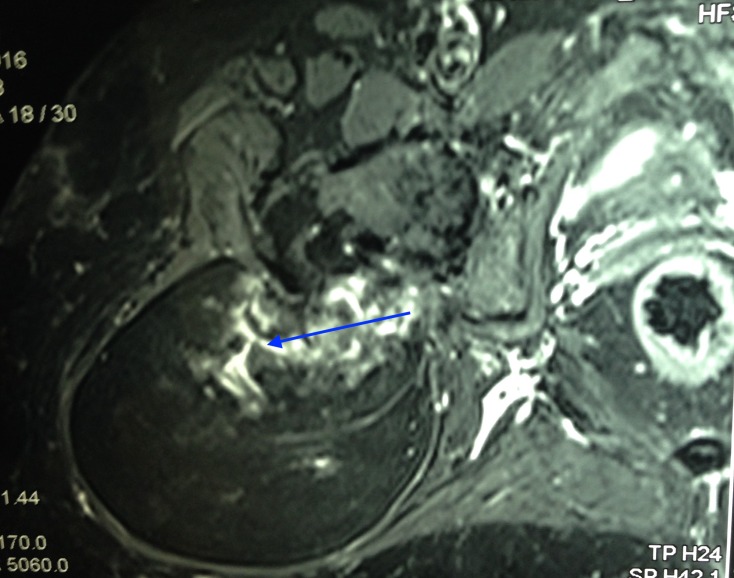
T2-weighted MRI showing tumour mass

An MRI revealed a well-circumscribed oval, lobular lipomatous mass that had high signal intensity on T1-weighted images (Figure 2) and low intensity signal on fat-suppressed T2-weighted images (Figure 3).

**Figure 4:**
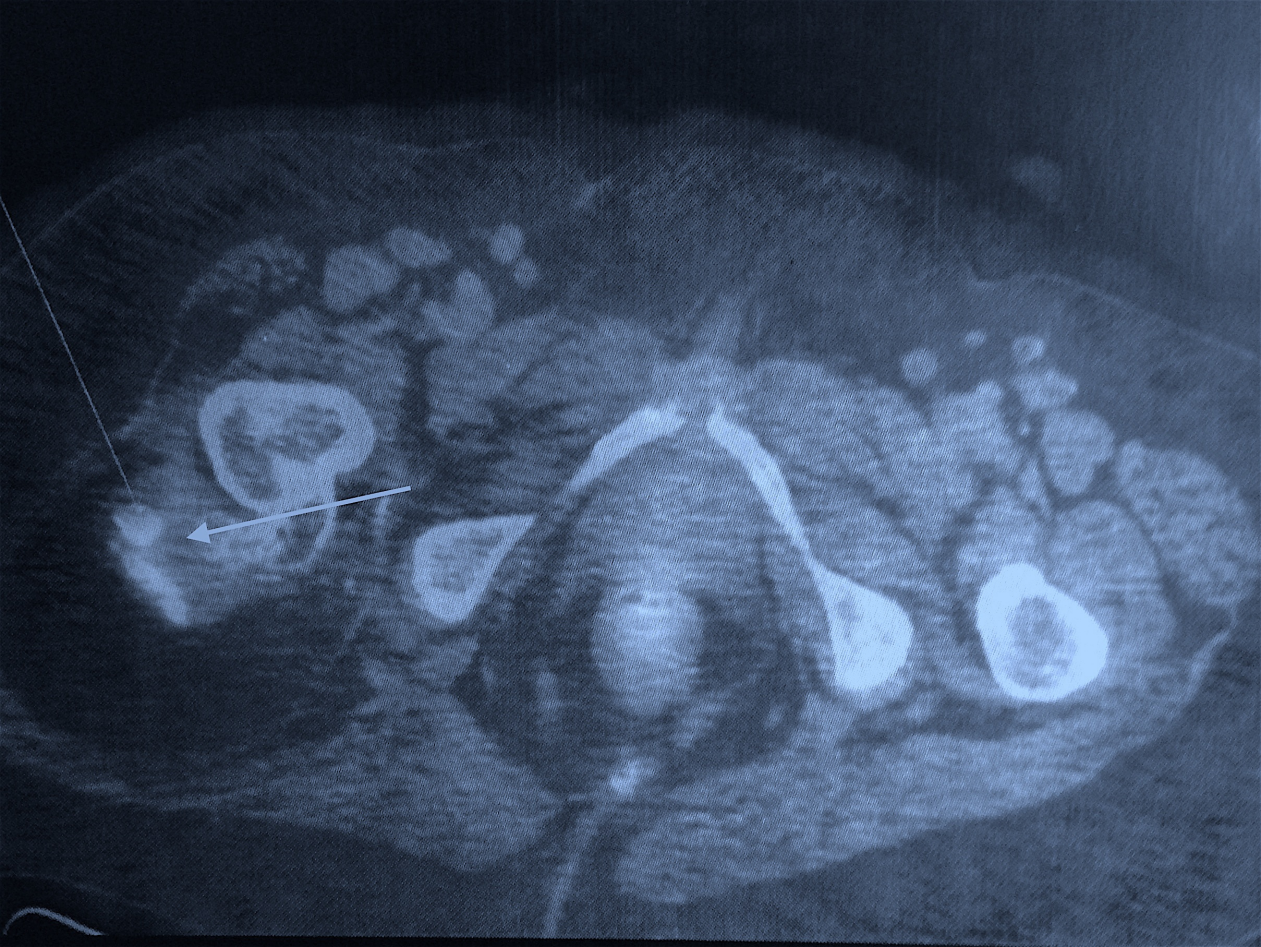
Fluoro-deoxy-glucose (FDG) bone scan showing a metabolically active lesion arising from the posterior aspect of the greater trochanter

The dense osseous tissue within the fatty core appeared as a hypointense cortical line on magnetic resonance imaging. A fluoro-deoxy-glucose (FDG) bone scan revealed a metabolically active lesion arising from the posterior aspect of the greater trochanter, showing chondroid type of calcifications within and low grade FDG uptake associated with a large soft tissue component showing fat density (Figure 4).

The preoperative differential diagnoses were primarily a lipoma with tumoral calcinosis, myositis ossificans, or an osteochondroma.

**Figure 5:**
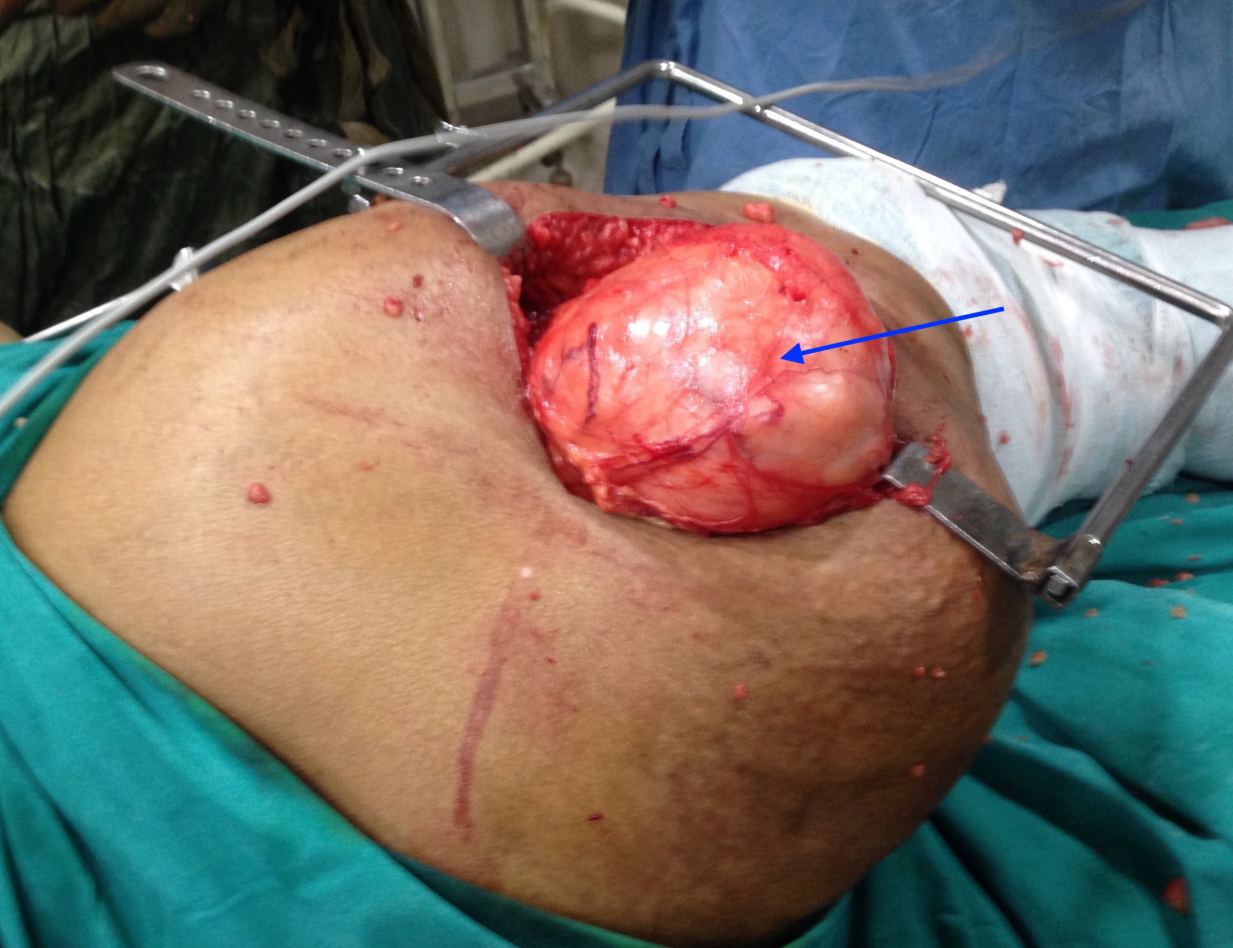
Intraoperative picture showing the tumour mass

An ultrasound-guided (USG) needle biopsy was performed revealing only benign mature fat cells and was evaluated as non-diagnostic. An excisional biopsy was undertaken. During the operation, a well encapsulated tumour mass was found to be located in the gluteal region underneath the gluteus maximus muscle (Figure 5).

**Figure 6:**
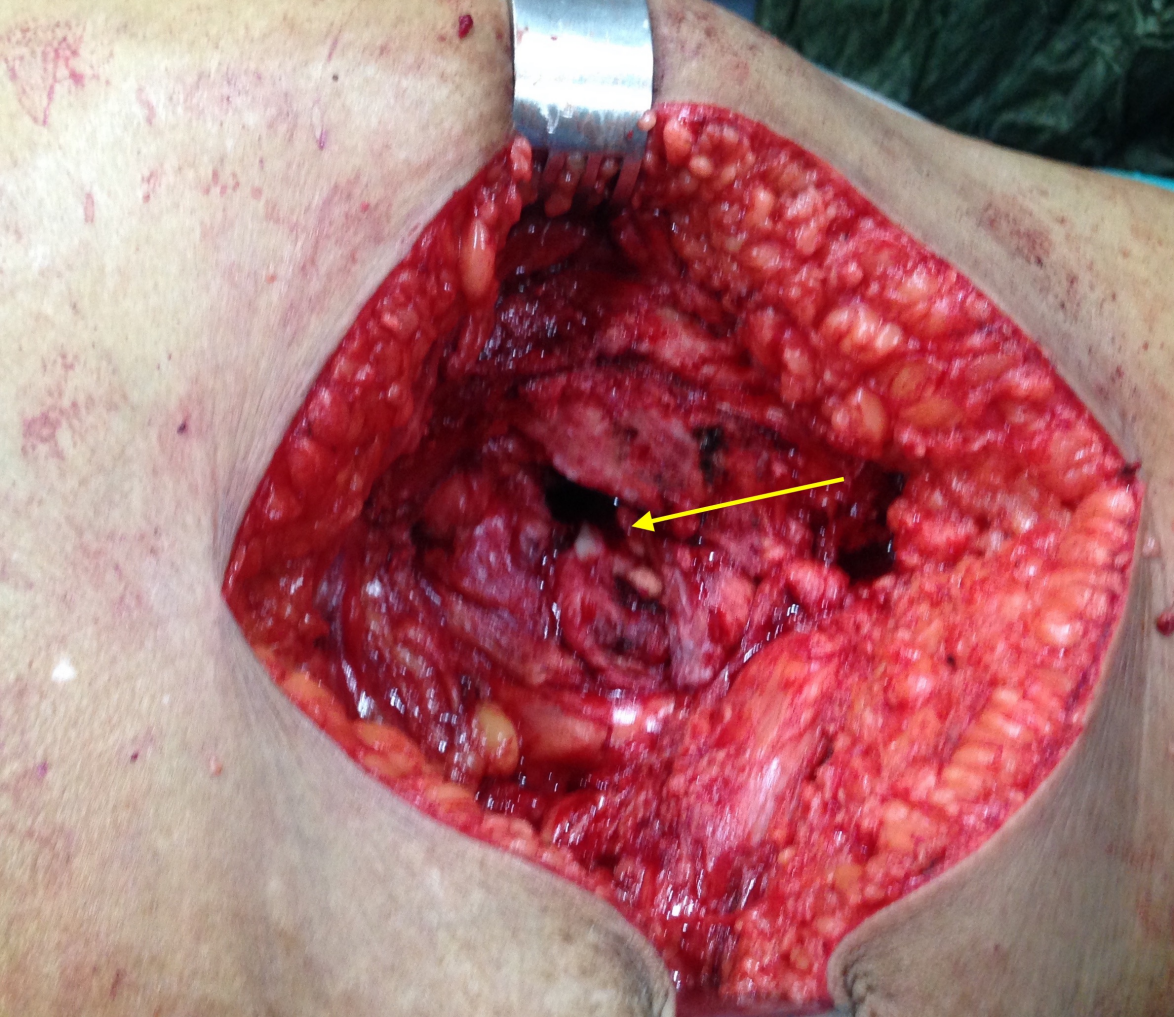
Intraoperative picture showing the basal (osseous) part of the tumour mass

At the base of the tumour, a basal osseous part was found arising from the periosteum of the greater trochanter of the femur. The tumour mass and the basal osseous part were removed surgically (Figure 6), and the incision was closed in four layers over a drain. Intraoperative blood loss was approximately 50 cc. The wound healed well, and the patient returned to daily activity after 10 days without complications.

**Figure 7:**
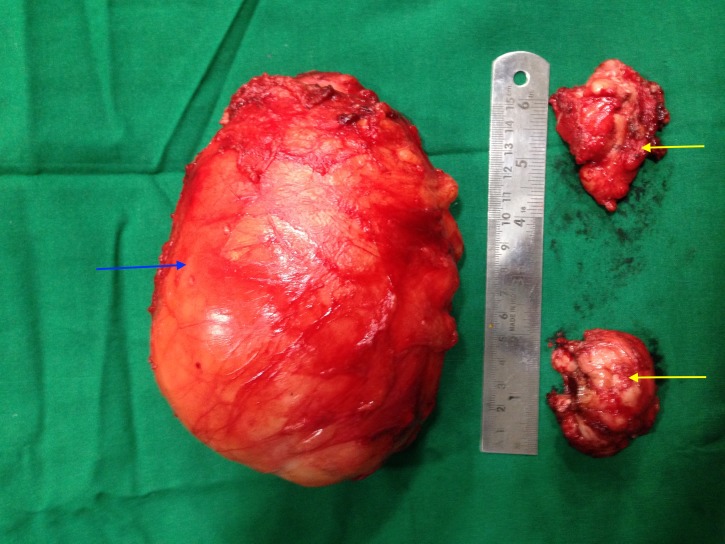
Excised lipomatous tumour (blue arrow) with basal osseous part (yellow arrow) (17 × 8 × 7 cm3)

Grossly, the resected specimen consisted of a mass measuring 17 × 8 × 7 cm^3^ with a smooth surface (Figure 7).

**Figure 8:**
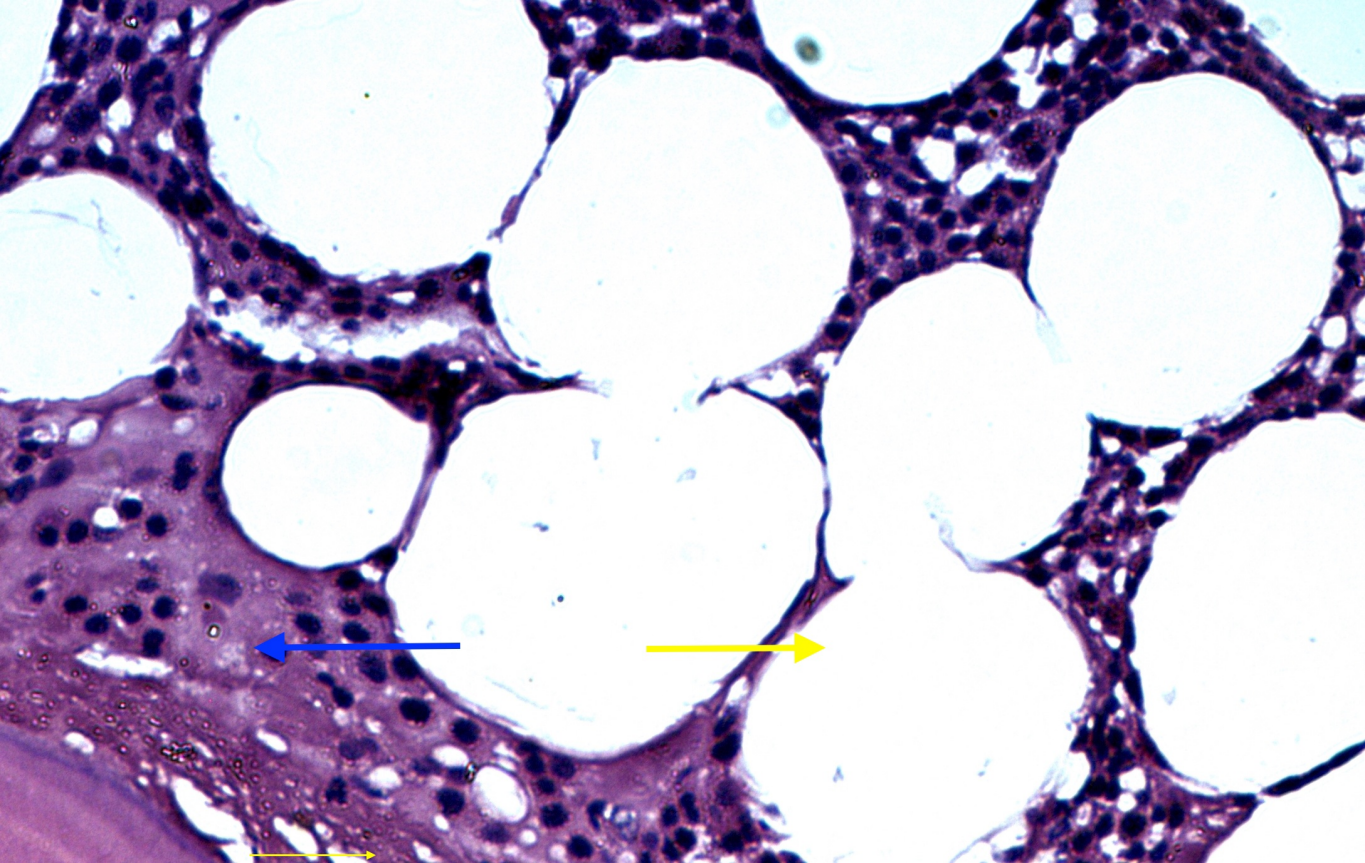
Microscopic view showing tumour mass consisting of mature adipose tissue (yellow arrow) surrounded by a thin layer of vascularized fibrous tissue (blue arrow)

Cut sections of the mass revealed mainly yellow fatty tissue surrounded by a thin fibrous capsule with numerous interlacing thin lamellar bony structures. Microscopically, the tumour consisted largely of mature adipose tissue surrounded by a thin vascular fibrous capsule. In regions with ossification, the bone was cortical type, composed of irregularly distributed interlacing lamellar and woven bone (Figure 8).

**Figure 9:**
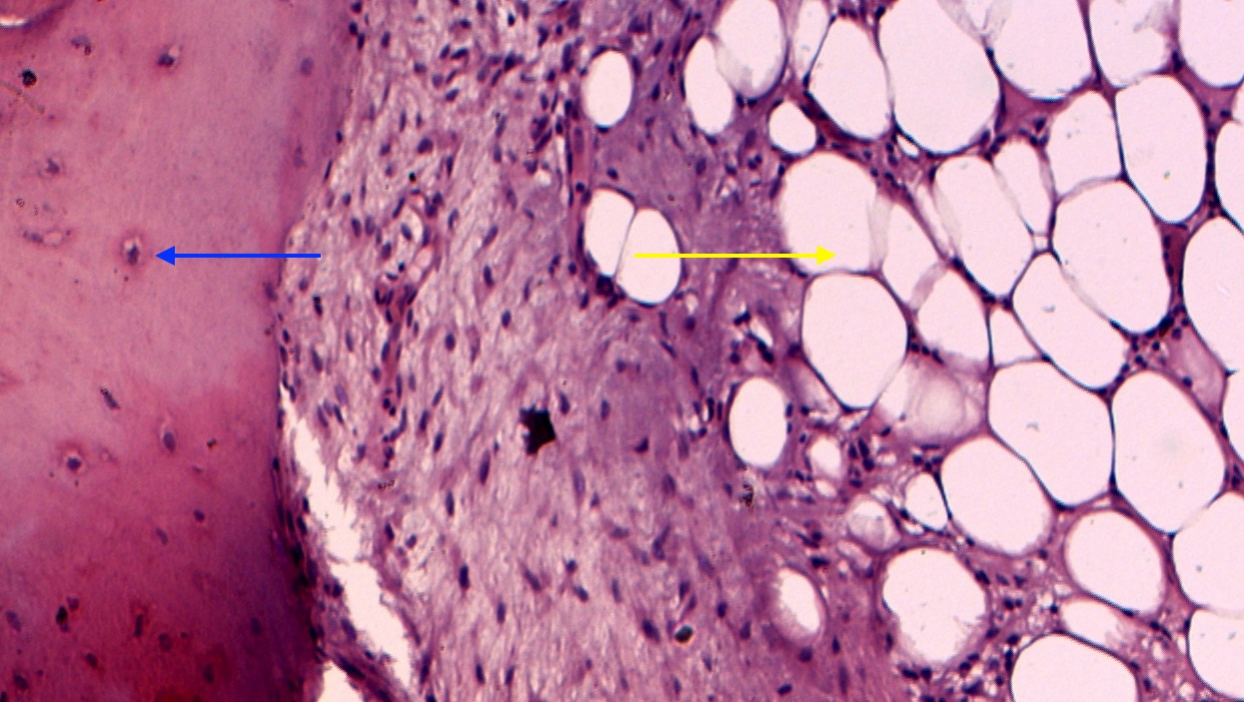
There was mature trabecular bone (blue arrow) within the mature adipose tissue (yellow arrow)

No myeloid cell islands were present. The adipocytes were uniform in size and shape with no cellular atypia, hypercellularity, mitotic figures, or necrosis (Figure 9).

The definitive pathological diagnosis was osteolipoma without evidence of malignancy. No recurrence was observed at eight months follow-up.

## Discussion

Lipomas are benign tumours containing mature adipose tissue. The lipomatous component is always predominant in these tumours. The World Health Organization classifies benign tumours of the adipose tissue into 14 types, depending on the presence of variable amounts of other mesenchymal tissue that form an intrinsic part of the tumour, such as fibrolipoma, myxoid lipoma, chondrolipoma, etc. Osseous metaplasia of a lipoma (osteolipoma) is rare.

The only previously reported case of osteolipoma in relation to the proximal femur was an intramuscular ossifying lipoma without evidence of malignancy in a middle-aged man [2]. In this case we report a giant osteolipoma arising from the periosteum of the greater trochanter of the femur in an elderly woman.

The pathogenesis of osteolipoma remains unclear, although two main theories exist. First theory: the tumour may originate directly from multipotent mesenchymal cells [2-3]. Second theory: the tumour may arise after repetitive trauma, ischemia, or metabolic changes leading to osseous metaplasia [4-5]. The osteoblastic activity is further strengthened by the environment of adipose tissue. Our histological findings and the indolent presentation of the tumour arising from the periosteum of the proximal femur in an elderly woman favor the second theory. A possible reason the osteolipoma grew adjacent to the greater trochanter is that repeated friction and microtrauma between the lipoma and perisosteum may have caused locally enhanced activity of osteoblasts on the periosteum, which subsequently led to ossification.

Imaging features of osteolipoma are pathognomonic [6-7]. A plain radiograph reveals dispersed calcifications close to the bone. This yields a differential diagnosis of a lipoma with tumoral calcinosis, myositis ossificans, or an osteochondroma. Magnetic resonance imaging usually reveals dense osseous tissue appearing as a hypointense cortical line within the lipomatous mass, which appears hyperintense on T1- and hypointense on T2-weighted images [8]. An FDG bone scan reveals a metabolically active lesion showing chondroid type of calcifications associated with a large soft tissue component showing fat density.

A definitive diagnosis is established with a histopathological examination. Microscopic appearance of mature adipose tissue with interlacing mature lamellar and woven bone in areas of ossification is the mainstay of the diagnosis [9]. The adipose tissue component is usually predominant and the mature bone tissue is irregular in distribution [9-10].

## Conclusions

Osteolipoma with an osseous basal portion is extremely rare. Surgical excision is the treatment of choice and the prognosis is good. Although osteolipomas are rare, it is necessary to have a high index of suspicion when encountering a lesion with adipose tissue in combination with ossification.
